# Having a child without wanting to? Estimates and contributing factors from a population-based survey in Sweden

**DOI:** 10.1177/1403494820965762

**Published:** 2020-11-06

**Authors:** Charlotte Deogan, Klara Abrahamsson, Louise Mannheimer, Charlotte Björkenstam

**Affiliations:** 1 The Public Health Agency of Sweden, Sweden; 2 Department of Global Public Health Sciences, Social Medicine, Infectious Diseases and Migration, Karolinska Institutet, Sweden; 3 Department of Public Health Sciences, Division of Learning, Informatics, Management and Ethics, Karolinska Institutet, Sweden; 4 Department of Neuroscience, Uppsala University, Psychiatry, Sweden

**Keywords:** Unintended pregnancy, unwanted pregnancy, unwanted children, fertility intentions, reproductive rights

## Abstract

**Aims:**

The aims of the current study were to identify the prevalence of unwanted childbirth (UC), to explore the association with sociodemographic factors and to identify possible contributing factors such as psychosomatic health, contraceptive use, experiences of induced abortion and sexual violence.

**Methods:**

We used Swedish data from the randomised population-based study SRHR2017 on sexual and reproductive health and rights (SRHR), based on self-administered surveys, linked to nationwide registers. The national sample consisted of 14,537 women and men aged between 16 and 84 years. With logistic regression, we examined differences in self-reported experience of UC, stratified by sex, in relation to socio-economic factors, as well as several possible contributing factors.

**Results:**

Despite advances in SRHR and fertility control, 6% of women and men in Sweden reported UC. This experience tends to be unevenly distributed in the population according to age, country of birth and, to some extent, income and educational attainment. Previous experience of induced abortion, sexual violence and threat from a partner were significantly associated with UC, whereas self-reported good health was protective.

**Conclusions:**

**Mechanisms behind unintended, unplanned, unwanted or mistimed pregnancies are complex. Current results focus on the role of individual factors and personal experiences. In addition, in line with previous understanding, there is a need for adopting a broader socio-ecological perspective on fertility intentions.**

## Introduction

The right to ‘decide whether, when and by what means to have a child or children, and how many children to have’ is essential to attaining sexual and reproductive health, which in turn is essential for public health and societal welfare [1]. Having control over one’s reproductive life is dependent on, for example, the right to information, access to effective contraception and safe abortion services and a life free from violence. Analysing experiences of unwanted childbirth (UC) helps us to understand underlying causes and to target support.

The terms ‘unintended’, ‘unplanned’ or ‘mistimed’ pregnancies are often used when measuring pregnancy intentions, but the term ‘unwanted’, in relation to both pregnancies and subsequent childbirth, is seldom explored. The definition of UC is still not clear, since research suggests it can overlap with unintended, unplanned or mistimed pregnancies. However, in this study, we use the term ‘unwanted childbirth’ as actually having had a child without wanting to. By using data from the cross-sectional randomised population-based study SRHR2017, we sought to explore UC in the Swedish population.

### Prevalence of unintended pregnancies and UC

Estimates from 2015 state that 90% of all European women who wanted to avoid conception were using contraceptives [2]. Yet, rates of unintended pregnancies are high. Studies suggest that 35% of all pregnancies in Western Europe and 54% in Eastern Europe are unintended [3]. A British population-based study indicated an annual prevalence of unintended pregnancies of 1.5% [4]. However, when measuring unintended pregnancies, answers largely depend on how and when questions are posed [5]. Rates of induced abortions cannot be used as a proxy of unintended pregnancies, since numbers globally tend to be underestimated. US data suggest that 42% of all unintended pregnancies end in induced abortions, and 44% in live births [6].

Having a child unwillingly may or may not intertwine with unwanted pregnancy, and can extend beyond the mother. Direct causality between unintended pregnancies, unintended births and subsequent health outcomes for the mother, child or family is difficult to demonstrate, even in longitudinal studies. Studies, however, suggest that antenatal maternal behaviours mediate the relationship between pregnancy intention and health outcomes [7]. Unwanted or mistimed pregnancies (and subsequent childbirth) have been associated with prenatal and post-partum behaviours such as seeking antenatal care at a later stage [7], smoking, alcohol and drug consumption [7,8], (post-partum) depression and anxiety [7,9] and a shorter period of breastfeeding [7], which can have a negative effect on the child, such as preterm births and low birthweight.

### The Swedish setting

For several reasons, we would expect rates of UC to be relatively low in Sweden. Previous studies have suggested that universalist welfare states, such as that in Sweden, provide good opportunities for people to translate intentions of childbearing into corresponding actions. Sweden is a forerunner on gender equality, with generous family policies [10]. There is universal access to sex education, contraceptive counselling, contraceptives and induced abortion that have been legal since 1973.

However, the unmet need for contraception among Swedish women has been estimated to be 8.9% according to a nationwide survey [11]. One reason could be the use of less effective methods instead of so-called long-acting reversible contraception. Furthermore, rates of pregnancy termination are higher in Sweden compared to the majority of other Western countries: around 35,000–38,000 annually (20/1000 women) in the those aged 15–44 years [12].

### Measuring UC

Previous studies have mainly focused on women, and concluded that not all women have a time plan for conception. Qualitative studies suggest that in order to formulate a pregnancy as ‘planned’, several criteria, such as stopping contraception, partner agreement and timing in relation to lifestyle and life stage, have to be met [13]. The terms ‘intended’, ‘wanted’ and ‘planned’ might carry different meanings for different respondents. A range of respondent options has been suggested to capture the phenomenon.

### Possible underlying mechanisms

The decision to have children is personal, but it is influenced by macro-level factors such as the welfare state and cultural changes in society. Religious and cultural beliefs or opposition, such as not accepting contraceptives or induced abortions [5], values and norm systems will also have an impact. Pressure and social control from parents, partners, peers and the community are also possible determinants of UC, especially in non-Western cultures [14]. Individual preferences might be difficult to disentangle from societal factors.

Individual determinants of unintended pregnancies are: length of the reproductive span, exposure to the risk of conception, the desired number of children, contraceptive use and effectiveness [5], subjective norms about childbearing and perceived behavioural control [10]. Early sexual debut, poor negotiation skills, high alcohol and drug use and low socio-economic status have also been identified as risk factors for unintended pregnancies [4]. Pregnancy intentions do not automatically translate into actions. Studies have shown that almost half of pregnancies reported as unintended occurred among women who were not using contraceptives [6].

US data have shown that those reporting an unwanted birth were more likely to be older [15] and socio-economically disadvantaged [15,16]. Access to reproductive health-care services and information, including induced abortion [5], are central determinants. Studies have also found that relational and economic uncertainty and a lack of consistency in the behaviours necessary to avoid unintended pregnancies (self-efficacy) were associated with unintended pregnancies [16].

Some evidence also points parents labelling pregnancies as unwanted when children have attributes that do not match the parents’ wishes. Some evidence for a preference for a mixed-sex composition was found in a study including European countries [17].

Studies conducted in both European middle- and high-income settings [18] and in middle- and low-income settings worldwide [19] found an association between experiences of abusive relationships (emotional, physical and/or sexual) and unintended pregnancies. In a European setting, experiences of abusive relationships were most common among those with a short education who were suffering economic hardship and had an ethnic background different from the majority in their country of residence [18].

In sum, UC is a public-health problem, and the prevalence of unwanted pregnancy remains high in the Western world, despite good access to contraceptives and counselling. Less is known on how common it is to have had a child unwillingly.

### Aims

The aim of the current study was to identify the prevalence of having had a child unwillingly. Furthermore, it aimed to explore differences in these experiences according to sociodemographic factors, and to analyse possible contributing factors.

## Methods

### Sample and data collection

The present study is based on a stratified randomised population-based survey on sexual and reproductive health and rights (SRHR), including Swedish residents aged 16–84 years, and performed by the Public Health Agency of Sweden in 2017. A sample of approximately 50,000 individuals were invited to participate in the survey, answering either online or by post during the autumn of 2017. The sampling of participants was based on information from the Swedish Total Population register, which includes information on all Swedish residents such as date of birth, age, sex, immigration dates, emigration dates and place of residence. The sampling frame consisted of 7,906,368 individuals. A simple stratified random sample of 50,016 individuals was drawn. Due to over-coverage, 232 individuals were excluded. Thus, 49,784 remained and received the questionnaire. In total, 15,186 individuals responded, generating a response rate of 31%. Non-responders were more likely to be men, young and born outside of Sweden and to have a lower educational level. The partial non-response varied between 0% and 14%. A total of 639 questionnaires were excluded due to contradictory responses, and another 582 questionnaires were excluded due to missing responses. Thus, the final sample consisted of 13,965 individuals. The sample was weighted to account for non-response on basis of sex, region of residence, country of birth and highest attained educational level. The SRHR2017 was further enriched by linking it to the national Longitudinal Integration Database for Health Insurance and Labor Market Studies (LISA). From LISA, information on sex, age, country of birth, region of residence, immigration status, highest attained educational level and income was obtained. Thus, none of the register-based variables had any missing values. The ethical committee in Stockholm approved the SRHR2017 study (Dnr: 2017/1011-31/5).

### Variables

#### Background variables

The following sociodemographic variables were included in the analyses: sex, age group (16–29, 30–44, 45–64 and 65–84 years), country of birth (Western countries and non-Western countries), region of residence (northern, mid-west, middle, Stockholm, south and south-west), highest attained educational level (⩽9 years, 10–12 years and >12 years) and income level (lowest income group (0–20) represents the 20% of individuals with lowest income, and highest income group (80–100) represent the 20% of individuals with the highest income).

#### Unwanted pregnancy

The outcome variable was based on the question ‘Have you ever had children even though you did not want to?’, with the response options ‘Yes’/‘No’.

#### Exposure variables

The outcome variable was examined in relation to health variables: psychosomatic health complaints (defined as having had headache, stomach ache, back pain, feeling low, feelings of irritation, bad mood, nervousness, experienced sleeping problems, dizziness or feeling powerless at least once a week during the past six months) and general health status (very good, good, neither good nor bad, bad or very bad), where the response alternatives ‘very good’ and ‘good’ were combined as ‘good’.

Lifetime use of ‘unsafe’ versus ‘safer’ contraceptive methods, by either the respondent or a partner, was also explored. Sexual intercourse interrupted by withdrawal of the penis before ejaculation, fertility computer or fertility apps and ‘safe periods’ were defined as ‘unsafe’ methods. Contraceptive pills (with oestrogen – combined), mini-pills, long-acting reversible contraception (contraceptive implants, contraceptive vaginal rings, contraceptive patches, contraceptive injections, hormonal coils), copper coils, condoms, diaphragms and emergency contraceptive pills were defined as ‘safer’ methods. Experiences of abortion was based on the question ‘Have you or a partner of yours ever been pregnant’, with the response options ‘Yes’/‘No’. Respondents answering ‘yes’ were then asked, ‘Has any pregnancy been terminated with an abortion?’, with the response options ‘Yes’/‘No’.

Experience of sexual violence was based on the question ‘Have you been subjected to (a) experiences of physical violence or threats of physical violence in an attempt to have sexual intercourse or perform similar sexual acts, or (b) physical violence or threats of physical violence in order to have forced sexual intercourse (oral, vaginal or anal)?’. Threat from a partner was based on the question ‘Have you had partners who have threatened to, for example, hurt themselves or the children, take the children and leave you, break your things, tell others about things that you wanted to keep secret?’

### Statistical analysis

Background demographics are presented by sex, using design information and sample weights. Second, we present proportions of having had children unwillingly stratified by sex. Third, with logistic regression analyses, we examined differences according to age, educational level, income level, geographical region and country of birth of having had a child without wanting to stratified by sex. We present three models: one crude, one adjusted for sex, age and country of birth and one additionally adjusted for geographical region and highest attained educational level. All confidence intervals were estimated with 95% certainty. All analyses were carried out using Stata v15 (StataCorp, College Station, TX).

## Results

### Estimates of ever having had a child without wanting to

In Table I, background demographics are presented as unweighted and weighted percentages. Table II presents estimates of the proportions who reporting having had children without wanting to in relation to background variables. A total of 6.1% (5.6–6.6%) reported having had a child without wanting to among individuals aged 16–84 years: 6.3% among women and 5.9% among men.

**Table I.** Sample characteristics by sex, unweighted and weighted (%).

	Unweighted		Weighted	
	Female	Male	Female	Male
*N*	7.903	6.062		
*Age (years)*				
16–29	24	16	21	22
30–44	25	21	24	25
45–64	26	28	32	32
65–84	26	35	23	21
*Country of birth*				
Western countries	97	96	91	91
Non-Western countries	3	4	9	9
*Educational level (years)*				
⩽9	13	16	17	20
10–12	37	42	43	46
>12	50	42	40	34
*Income level*				
0–20	22	17	22	18
20–40	20	20	20	20
40–60	19	22	18	22
60–80	20	20	20	20
80–100	20	21	20	21
*Geographical region*				
Northern	18	17	9	9
Mid-west	16	17	23	23
Middle	17	16	15	15
Stockholm	17	16	24	23
South	15	17	13	13
South-west	17	17	16	17

**Table II.** Proportions who have had children without wanting to, women and men.

	Women	Men
*Age (years)*		
16–29	2.4 (1.6–3.6)	1.7 (1.0–2.7)
30–44	5.8 (4.5–7.4)	6.5 (5.0–8.4)
45–64	6.5 (5.3–7.9)	7.9 (6.6–9.5)
65–84	8.8 (7.5–10.3)	8.2 (6.9–9.7)
*Country of birth*		
Western countries	5.5 (4.9–6.1)	5.7 (5.0–6.4)
Non-Western countries	11.0 (7.5–15.8)	12.2 (8.5–17.2)
*Educational level (years)*		
⩽9	7.5 (5.8–9.6)	4.1 (3.0–5.7)
10–12	7.0 (5.9–8.2)	6.9 (5.9–8.2)
>12	4.8 (4.0–5.9)	7.2 (6.0–8.7)
*Income level*		
0–20	7.9 (6.5–9.6)	8.4 (6.5–10.8)
20–40	6.8 (5.4–8.5)	5.6 (4.3–7.2)
40–60	6.7 (5.2–8.5)	7.2 (5.7–9.0)
60–80	4.6 (3.4–6.3)	4.2 (3.1–5.7)
80–100	4.0 (2.9–5.3)	6.2 (4.8–8.1)
*Geographical region*		
Northern	4.6 (3.5–6.0)	4.8 (3.5–6.4)
Mid-west	5.8 (4.5–7.5)	6.1 (4.8–7.8)
Middle	5.8 (4.5-7.5)	5.1 (3.9–6.8)
Stockholm	7.1 (5.7–9.0)	8.2 (6.4–10.5)
South	5.3 (3.9–7.0)	6.0 (4.5–8.0)
South-west	6.0 (4.6–7.8)	5.8 (4.4–7.5)

Data shown as % with 95% confidence intervals (CI).

Education was the only background variable in which statistically significant sex differences were found. Women with less than nine years of education more often reported experiences of having had children without wanting to (7.5%, 95% CI 5.8–9.6%) compared to men (4.1%, 95% CI 3.0–5.7%). The difference was reversed among those with more than 12 years of education (women: 4.8%, 95% CI 4.0–5.9%; men: 7.2%, 95% CI 6.0–8.7%). Naturally, there were significant differences across age groups for both women and men, as having a child without wanting to was more common with increasing age.

Estimates were higher for those born in non-Western countries compared to those born in Western countries, including Sweden:11.0% (95% CI 7.5–15.8%) compared to 5.5% (95% CI 4.9–6.1%) among women, and 12.2% (95% CI 8.5–17.2%) compared to 5.7% (95% CI 5.0–6.4%) among men.

Among women, there was an income gradient, in which proportions of unwanted pregnancies were more commonly reported among women with the lowest income (7.9%, 95% CI 6.5–9.6%) compared to women with the highest income (4.0%, 95% CI 2.9–5.3%). Among men, there was no such gradient, even though proportions were highest among men with the lowest income: 8.4% (95% CI 6.5–10.8%).

In the logistic regression models, the odds of having had a child without wanting to were highest in the oldest age group (65–84 years) for both women and men, after adjusting for several background factors (model A and B, Table III).

**Table III.** Risk of having had a child without wanting to in relation to sex, age, country of birth, geographical region, income level and educational level (model 1).

	All		Women		Men		
	Crude	Crude	Model A^a^	Model B^b^	Crude	Model A^a^	Model B^b^
*Sex*							
Female	1 (ref)						
Male	1.05 (0.89–1.24)						
*Age (years)*							
16–29	1 (ref)	1 (ref)	1 (ref)	1 (ref)	1 (ref)	1 (ref)	1 (ref)
30–44	3.15 (2.18–4.55)	2.49 (1.54–4.04)	2.37 (1.46–3.84)	2.31 (1.38–3.88)	4.07 (2.32–7.17)	3.82 (2.17–6.71)	2.92 (1.62–5.26)
45–64	3.74 (2.64–5.29)	2.83 (1.79–4.47)	3.11 (1.97–4.92)	2.80 (1.72–4.56)	5.02 (2.95–8.57)	5.36 (3.11–9.25)	4.14 (2.36–7.28)
65–84	4.46 (3.18–6.26)	3.91 (2.51–6.01)	4.47 (2.87–6.95)	3.62 (2.31–5.68)	5.20 (3.06–8.83)	5.79 (3.36–9.99)	4.71 (2.71–8.2)
*Country of birth*							
Western countries	1 (ref)	1 (ref)	1 (ref)	1 (ref)	1 (ref)	1 (ref)	1 (ref)
Non-Western countries	2.23 (1.63–3.01)	2.12 (1.38–3.29)	2.69 (1.72–4.2)	2.67 (1.66–4.28)	2.31 (1.52–3.53)	2.70 (1.75–4.16)	2.87 (1.85–4.45)
*Geographical region*							
Mid-west	1 (ref)	1 (ref)		1 (ref)	1 (ref)		1 (ref)
Northern	0.78 (0.59–1.03)	1.00 (0.68–0.45)		0.94 (0.64–1.37)	0.77 (0.51–1.15)		0.86 (0.57–1.28)
Middle	0.91 (0.69–1.19)	0.79 (0.54–1.15)		0.81 (0.55–1.2)	0.83 (0.56–1.23)		0.71 (0.47–1.08)
Stockholm	1.31 (1.00–1.70)	1.25 (0.87–1.79)		1.29 (0.89–1.86)	1.37 (0.94–2.00)		1.31 (0.89–1.93)
South	0.94 (0.71–1.25)	0.90 (0.60–1.35)		0.86 (0.57–1.29)	0.97 (0.65–1.46)		0.90 (0.59–1.37)
South-west	0.98 (0.52–0.76)	1.03 (0.70–1.53)		1.03 (0.69–1.53)	0.94 (0.64–1.37)		0.93 (0.63–1.38)
*Income level*							
0–20	1 (ref)	1 (ref)			1 (ref)		
20–40	0.75 (0.58–0.96)	0.85 (0.62–1.18)			0.64 (0.43–0.95)		
40–60	0.85 (0.66–1.09)	0.83 (0.59–1.17)			0.84 (0.59–1.23)		
60–80	0.52 (0.39–0.70)	0.57 (0.39–0.84)			0.48 (0.32–0.73)		
80–100	0.61 (0.46–0.80)	0.48 (0.33–0.70)			0.72 (0.49–1.07)		
*Educational level (years)*							
⩽9	1 (ref)	1 (ref)		1 (ref)	1 (ref)		1 (ref)
10–12	1.24 (0.97–1.58)	0.93 (0.68–1.28)		0.92 (0.65–1.31)	1.73 (1.18–2.55)		1.60 (1.07–2.40)
>12	1.04 (0.81–1.34)	0.63 (0.45–0.88)		0.58 (0.4–0.85)	1.80 (1.21–2.68)		1.46 (0.96–2.22)

Data shown as odds ratios (OR) with 95% CI.

a Adjusted for: sex, age and country of birth.

b Adjusted for sex, age, country of birth, geographical region and educational level.

Being born in non-Western countries was positively associated with the outcome and among both women (multi-adjusted odds ratio (OR)=2.67, 95% CI 1.66–4.28) and men (multi-adjusted OR=2.87, 95% CI 1.85–4.45).

After adjustments, the risk of having had a child without wanting to decreased with an increase in income. However, the relationship was not linear. In the highest income category, the risk of having a child unwillingly was 0.48 (95% CI 0.33–0.70) for women and 0.72 (95% CI 0.49–1.07) for men.

For men, having more than 12 years of education increased the odds of having had a child unwillingly (multi-adjusted OR=1.54, 95% CI 1.02–2.34) compared to men with no more than nine years of education. For women, the opposite was true, as a longer education decreased the odds (multi-adjusted OR=0.57, 95% CI 0.39–0.83).

As described in Table IV, psychosomatic health complaints were positively associated with the outcome (multi-adjusted OR=1.37, 95% CI 1.15–1.63), and reporting good or very good health was negatively associated with the outcome (multi-adjusted OR=0.69, 95% CI 0.57–0.84).

**Table IV.** Risk of having had a child without wanting to in relation to contraceptive use and experiences of induced abortion, sexual violence and threat from partner and health complaints and general health status.

	Crude	Multi-adjusted^a^
Good general health	0.62 (0.52–0.74)	0.69 (0.57–0.84)
Psychosomatic health complaints	1.20 (1.01–1.42)	1.37 (1.15–1.63)
Sexual violence	1.84 (1.39–1.83)	1.80 (1.34–2.42)
Threat from partner	2.64 (2.10–3.32)	2.99 (2.34–3.82)
Abortion	3.04 (2.53–3.66)	3.12 (2.57–3.81)
*Contraception*		
Safer	1.60 (1.11–2.33)	1.68 (1.15–2.46)
Unsafe	2.26 (1.56–3.29)	2.63 (1.78–3.88)

Data shown as OR with 95% CI.

a Adjusted for: sex, age, country of birth, geographical region and income.

Among individuals who reported employing ‘safer’ methods of contraception, the odds were 1.68 higher (multi-adjusted OR, 95% CI 1.15–2.46) compared to those who had not used any method of contraception. Among those employing ‘unsafe’ methods of contraception, the odds were 2.63 higher (multi-adjusted OR, 95% CI 1.78–3.88) compared to non-users (Table IV). The odds were also threefold higher among those who had experienced induced abortion, whether as pregnant or as a partner, any time in life (multi-adjusted OR=3.07, 95% CI 2.52–3.74).

Furthermore, there was a strong association between having had a child without wanting to and experiences of both threat from a partner (multi-adjusted OR=3.0, 95% CI 2.34–3.82) and sexual violence (multi-adjusted OR=1.80, 95% CI 1.34–2.42; Table IV).

## Discussion

### Findings and interpretation in relation to previous studies

Increasing outcome prevalence along with increasing age is natural in a questionnaire that asks for lifetime prevalence. Furthermore, a stark bias is of course that the oldest respondents did not have access to legal induced abortion during their fertile years. The lower prevalence among the younger groups can also be due to medical advances, such as access to legal induced abortion, but also relate to changed norms and attitudes concerning childrearing and family formation.

It appears as if a longer education could function as a protective factor for women’s reproductive decisions and autonomy. Educational attainment may contribute through both selective and causal mechanisms. Women from poorer families may have less access to reproductive health care early in life, and thus may be more likely to face the same challenges later in life. In addition, the perceived costs of having a child might be less among women with lower education, and unwanted births may derail women’s future educational trajectories. For men, there might be a selection effect in which well-educated men are seen as more attractive on the ‘marital market’ and therefore more likely to become fathers, both intended and unintended. There are Swedish research findings that support the idea that less educated men are more likely to become involuntarily single and childless [20]. Previous research has suggested that early parenthood is associated with less sense of autonomy and control and perceiving life as less pleasurable and meaningful [21]. These factors were largely mediated by later life socio-economic and health status.

Being born in non-Western countries remained positively associated with the outcome for both women and men. This indicates the underlying differences in vulnerability and deprivation, depending on cultural and social background.

Among those reporting using both ‘safer’ and ‘unsafe’ methods of contraception, the odds of having had children without wanting to were elevated, but more so among those employing only ‘unsafe’ methods. Since we lack information on motivation and reasons behind contraceptive use, we cannot fully explain this difference. Some of those who did not use any contraceptive method at all (unsafe) had not had sex during the past 12 months (11%). The odds of having had a child unwillingly were three times higher among individuals with experience of induced abortion. Many young women do not use contraceptives in between partners, and these numbers are higher in Sweden as compared to Finland for instance [11].

Psychosomatic health complaints were associated with having had children unwillingly in the present study. This is in line with a review of the literature from 2013, which demonstrates that births resulting from unintended pregnancy were associated with elevated levels of depression and anxiety and lower reported levels of happiness [7].

The finding that experiences of sexual violence were associated with higher odds of having had children without wanting to has been demonstrated previously [18]. Violence, whether within a relationship or not, might limit reproductive freedom and decision making. The feeling of not wanting a child might be an expression of not wanting a child within that particular relationship. However, we do not know whether the sexual violence occurred in proximity to the unwanted pregnancy, since both questions relate to lifetime experiences.

### Strengths and limitations

SRHR2017 provides unique data with nationally representative population data, including both women and men, enriched with high-quality nationwide register data. Our study does, however, have several limitations. The use of cross-sectional data precludes any causal interpretation regarding the relationship between having had a child unwillingly and any associated factors. Our results therefore generate some further hypotheses rather than conclude causal associations. Furthermore, the response rate was 31%. However, the sample was weighted to account for non-response which helps us ascertain representability. Yet, it remains unclear exactly how the lower response rates from foreign-born respondents may have impacted the prevalence of having a child without wanting to. Also, we only measured the occurrence of UC with one item. Some studies adopt a set of questions: if the pregnancy was intended (i.e. wanted at the time), mistimed (wanted but not at the time it occurred) or unwanted (not wanted at any time) [18]. Perhaps numbers would have been higher if the question had been posed in a more open manner, since the term ‘not wanting to’ is quite strong compared to ‘not planning to’ or ‘not intending to’, which indicates our estimates of having a child without wanting to are an underestimation of unintended or mistimed pregnancies. Last, having a child without wanting to was captured retrospectively. We do not know if some of the exposures occurred prior to or after the outcome. Previous studies have recommended retrospective measurement when assessing unwanted fertility, as numbers are more accurate in relation to the actual number of births [15]. There are, however, issues with both prospective and retrospective strategies when measuring unwanted fertility [22]. First, there is the risk of recall bias, in which the respondent might not remember if they wanted children at the time of the pregnancy. Second, rationalisation bias or social desirability bias might occur, in which pregnancies that end with childbirth in retrospect are less likely to be reported as unwanted. The fact that not even 1% of the respondents in SRHR2017 reported having more children than they had intended or wished for points to the previously noted finding [22] that existing children are unlikely to be reported as unwanted, even in cases where a current pregnancy is referred to as mistimed or unwanted. Underlying mechanisms include social, religious and cultural control and regulation ofsexual and reproductive lives, especially women’s, by families, communities and societies [14,23].

### Future interventions and research

Universal as well as directed interventions on fertility planning are encouraged, especially towards low-educated women. Strengthened sexual education and provision of good-quality health services for young adults, including asking about experiences of violence in prenatal care, are recommended. Secondary prevention interventions could include targeted parental support interventions to at-risk families. Studies have shown that mothers’ retrospective perceptions of pregnancy desire can be predictive for the child’s social-emotional development [24]. In the post-partum period, information on the child being wanted could be gathered by health-care personnel to guide them on where to focus resources.

More research needs to focus on men’s reproductive goals and needs. Partners’ influences on women’s fertility intentions could also be an area of investigation, as well as a more nuanced picture on fertility intentions, in which both the positive and negative consequences of having children should be highlighted.

## Conclusions

In our study, 6% of the Swedish population reported having had a child without wanting to. Lower income, being born in non-Western countries and shorter education for women and longer education for men were statistically significantly associated with having had a child without wanting to. The presence of psychosomatic health complaints, poor general health, ‘unsafe’ contraceptive use and experiences of threat from a partner and of sexual violence were all contributing factors.

## References

[1] Guttmacher-Lancet Commission on Sexual and Reproductive Health and Rights. Accelerate progress-sexual and reproductive health and rights for all. Executive summary. The Lancet; 2018 (Available from: https://www.thelancet.com/commissions/sexual-and-reproductive-health-and-rights.) 10.1016/S0140-6736(18)30293-9 29753597

[2] United Nations. Trends in Contraceptive Use Worldwide 2015. Department of Economic and Social Affairs, Population Division, https://www.un.org/en/development/desa/population/publications/pdf/family/trendsContraceptiveUse2015Report.pdf; 2015.

[3] Sedgh G Hussain R. Reasons for contraceptive nonuse among women having unmet need for contraception in developing countries. Stud Fam Plan 2014;45:414. 10.1111/j.1728-4465.2014.00382.x 24931073

[4] Wellings K , et al. The prevalence of unplanned pregnancy and associated factors in Britain: findings from the third National Survey of Sexual Attitudes and Lifestyles (Natsal-3). Lancet 2013;382:1807–16. 24286786 10.1016/S0140-6736(13)62071-1 PMC3898922

[5] ESHRE Capri Workshop Group. Why after 50 years of effective contraception do we still have unintended pregnancy? A European perspective. Hum Reprod 2018;33:777–83. 29659848 10.1093/humrep/dey089

[6] Finer LB Henshaw SK. Disparities in rates of unintended pregnancy In the United States, 1994 and 2001. Perspect Sex Reprod Health 2006;38:90–6. 16772190 10.1363/psrh.38.090.06

[7] Gipson JD Koenig MA Hindin MJ. The effects of unintended pregnancy on infant, child and parental health: a review of the literature. Stud Fam Plan 2008;39:18–38. 10.1111/j.1728-4465.2008.00148.x 18540521

[8] Joyce T Kaestner R Korenman S. The effect of pregnancy intention on child development. Demography 2000;37:83–94. 10748991

[9] Rackin HM Brasher MS. Is baby a blessing? Wantedness, age at first birth, and later-life depression. J Marriage Fam 2016;78:1269–84.

[10] Holland JA Keizer R. Family attitudes and fertility timing in Sweden. Eur J Popul 2015;31:259–85.

[11] Kopp Kallner H Thunell L Brynhildsen J , et al. Use of contraception and attitudes towards contraceptive use in Swedish women – a nationwide survey. PLoS One 2015; 10. 10.1371/journal.pone.0125990 PMC4439158 25992901

[12] Socialstyrelsen. Statistik om aborter. https://www.socialstyrelsen.se/globalassets/sharepoint-dokument/artikelkatalog/statistik/2020-6-6806.pdf; 2019.

[13] Barrett G Wellings K. What is a ‘planned’ pregnancy? Empirical data from a British study. Soc Sci Med 2002;55:545–57. 12188462 10.1016/s0277-9536(01)00187-3

[14] Sanjakdar F . Cultural constructions of sexuality and gender. In: Living west, facing east: the (de)construction of Muslim youth sexual identities. Counterpoints series. Vol. 364. New York: Peter Lang, 2011, pp.105–30.

[15] Rackin HM Morgan SP. Prospective versus retrospective measurement of unwanted fertility: strengths, weaknesses, and inconsistencies assessed for a cohort of US women. Demogr Res 2018;39:61–94. 31827372 10.4054/DemRes.2018.39.3 PMC6905391

[16] Musick K England P Edgington S , et al. Education differences in intended and unintended fertility. Soc Forces 2009;88:543–72. 23436948 10.1353/sof.0.0278 PMC3578704

[17] Hank K Kohler H-P. Gender preferences for children in Europe: empirical results from 17 FFS countries. Demogr Res 2000;2.

[18] Lukasse M Laanpere M Karro H , et al. Pregnancy intendedness and the association with physical, sexual and emotional abuse – a European multi-country cross-sectional study. BMC Pregnancy Childbirth 2015;15:120. 26008119 10.1186/s12884-015-0558-4 PMC4494794

[19] Pallitto CC García-Moreno C Jansen HA , et al. Intimate partner violence, abortion, and unintended pregnancy: results from the WHO multi-country study on women’s health and domestic violence. Int J Gynaecol Obstet 2013;120:3–9. 22959631 10.1016/j.ijgo.2012.07.003

[20] Boschini A Sundström M. Det ojämlika faderskapet. Ekonomisk Debatt 2018;4.

[21] Read S Grundy E. Fertility history and quality of life in older women and men. Ageing Soc 2011;31:125–45.

[22] Bachrach CA Newcomer S. Intended pregnancies and unintended pregnancies: distinct categories or opposite ends of a continuum? Fam Plan Perspect 1999;31:251–2. 10723654

[23] Furstenberg F. Destinies of the disadvantaged: the politics of teen childbearing. New York: Russell Sage Foundation, 2007.

[24] Saleem HT Surkan PJ. Parental pregnancy wantedness and child social-emotional development. Matern Child Health J 2014;18:930–8. 23793490 10.1007/s10995-013-1320-z PMC4437702

